# Supplementary material for: A disease-agnostic approach to ensemble learning for infectious disease forecasting
Source: Nat Commun. 2026 Mar 20;17:4255. doi: 10.1038/s41467-026-70937-8 (PMC13168361; doi:10.1038/s41467-026-70937-8)
Supplement: Supplementary file 1 — Supplementary Information [file 41467_2026_70937_MOESM1_ESM.pdf]

# SUPPLEMENTARY MATERIALS FOR “BEYOND EQUAL WEIGHTS: A DISEASE-AGNOSTIC APPROACH TO ENSEMBLE LEARNING FOR INFECTIOUS DISEASE FORECASTING"

CONTENTS

1 EpiFFORMA Up-weights Appropriate Component Models . . . . . . . . . . . . . . 2
2 Distribution of EpiFFORMA Ranks Compared to Equal Weights . . . . . . . . . . 2
3 Implementation Details . . . . . . . . . . . . . . . . . . . . . . . . . . . . . . . . 3
3.1 Synthetic Data Generation . . . . . . . . . . . . . . . . . . . . . . . . . . 3
3.2 Time Series Pre-Processing . . . . . . . . . . . . . . . . . . . . . . . . . . 5
3.3 Feature Extraction . . . . . . . . . . . . . . . . . . . . . . . . . . . . . . . 7
3.4 Component Forecast Modeling . . . . . . . . . . . . . . . . . . . . . . . . 8
3.5 GBM Meta-Model . . . . . . . . . . . . . . . . . . . . . . . . . . . . . . . 9
3.6 Equal Weights Ensemble . . . . . . . . . . . . . . . . . . . . . . . . . . . 10
3.7 Uncertainty Quantification . . . . . . . . . . . . . . . . . . . . . . . . . . 10
4 Data Sources and Cleaning . . . . . . . . . . . . . . . . . . . . . . . . . . . . . 12
4.1 COVID-19 . . . . . . . . . . . . . . . . . . . . . . . . . . . . . . . . . . . 12
4.2 ILI . . . . . . . . . . . . . . . . . . . . . . . . . . . . . . . . . . . . . . . 12
4.3 Dengue Fever . . . . . . . . . . . . . . . . . . . . . . . . . . . . . . . . . 12
4.4 Chikungunya (Brazil) . . . . . . . . . . . . . . . . . . . . . . . . . . . . . 13
4.5 Diphtheria, Measles, Mumps, Polio, Rubella, and Smallpox . . . . . . . . . . 13
5 Assessment of Synthetic Data Coverage . . . . . . . . . . . . . . . . . . . . . . 13
5.1 Dimensionality Reduction via Uniform Manifold Approximation and Projection (UMAP) . . . . . . . . . . . . . . . . . . . . . . . . . . . . . . . . 13
6 Analysis of epiFFORMA by Regimes . . . . . . . . . . . . . . . . . . . . . . . . 14
7 Comparison of epiFFORMA to alternative online ensembling techniques . . . . . . 16
References . . . . . . . . . . . . . . . . . . . . . . . . . . . . . . . . . . . . . . 16

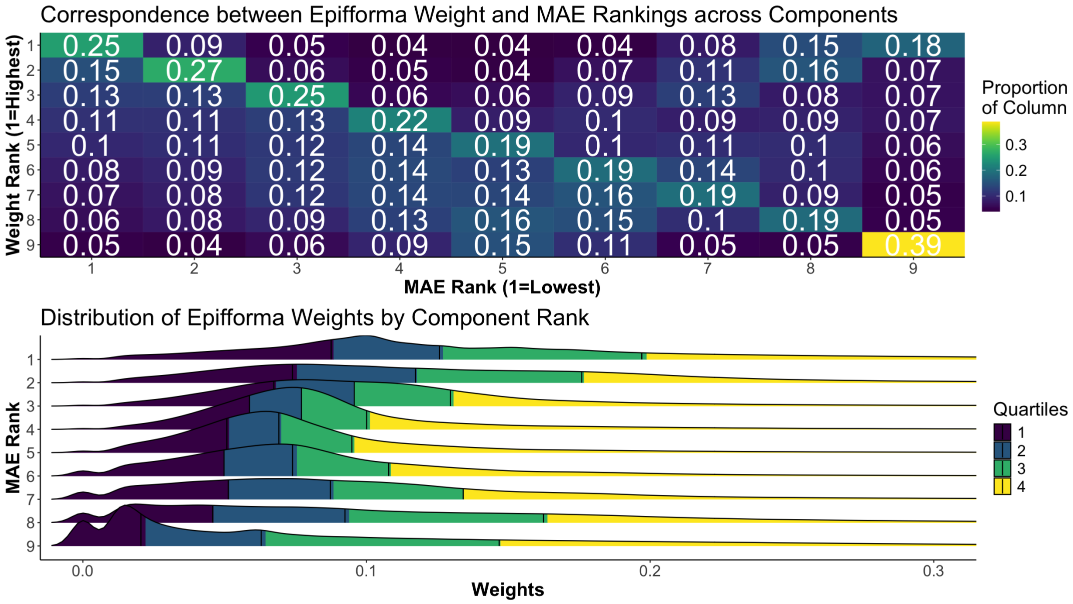


FIG S1. *Distribution of weights allocated to each component model according to a given model's forecasting accuracy, averaged over all data applications; ties are broken randomly. The columns of the figure on top all add to 1. EpiFFORMA tends to more highly weight the models with higher forecasting accuracy.*

**1. EpiFFORMA Up-weights Appropriate Component Models.** We argue that epiFFORMA performs as desired – giving higher weights to more accurate component models according to a given data scenario – across several locations and diseases. In Figure S1, we visualize the weight assignments by how well each component model performed compared to the other component models. Along the top of Figure S1, a single cell corresponds to the average number of times across all disease forecasts that a component model at a certain rank (according to its forecasting accuracy, along the bottom axis) was assigned a given rank by epiFFORMA (according to ensemble weight allocation, along the left vertical axis). For instance, the cell on the top-left hand corner describes the proportion of times that the component model that performed best (Rank = 1 according to MAE) for a given forecast was also given the highest weight (Weight Rank = 1) by epiFFORMA. Accurate weight assignments from epiFFORMA would correspond to higher relative proportions along the diagonal of this plot, which can be seen visually in top part of Figure S1. Note that there is also an above average proportion of the worst-performing model being given the highest-weighted position (the top-right cells). We suspect that this proportion comes from instances where epiFFORMA expects either a significant increase or decrease in cases, yet the opposite happens.

The distributions of all weights assigned to the models at different ranks of forecasting accuracy are visualized along the bottom of Figure S1. This figure shows that although there remain a wide range of weights assigned by epiFFORMA, the distributions of weights for the models that had higher performance are typically shifted to the right. This shows how epiFFORMA typically assigns higher weights to better component models.

**2. Distribution of EpiFFORMA Ranks Compared to Equal Weights.** In Figure S2, the distribution of rankings is directly compared to the equal-weighting forecasts across all data applications (not including any forecasts on the synthetic data). The equal-weighting forecasts were calculated by taking a simple mean of all component model forecasts except

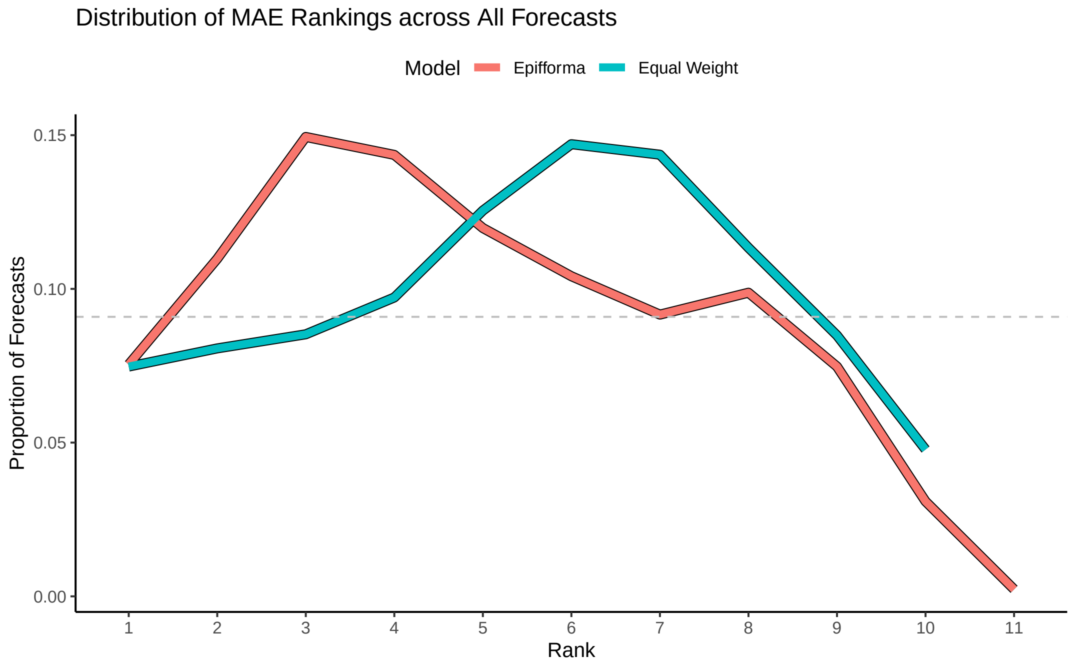


FIG S2. *The rank of epiFFORMA compared to the equal-weighting scheme and the individual component models for the diseases considered in Table 1. The overall average performance for each model is ranked (along the vertical axis), for each disease and for the synthetic data (along the horizontal axis). EpiFFORMA is more often the top-performing model over the* $n = 2021958$ *forecasts calculated for this experiment. The equal-weighting scheme excluded the mirror model.*

the mirror component model, which received no weight. The horizontal dashed line represents the scenario where the ranks of models are selected uniformly at random. This figure shows that although epiFFORMA may not always be the most accurate (i.e. top-ranked) forecast for an individual forecast, it is more often in the top 5 forecasts than the equal-weight ensemble. This is in-line with expectations.

**3. Implementation Details.** In this section, we provide detailed descriptions of the implementation of the epiFFORMA methodology used in the main paper. This section is partitioned into six primary subsections, each covering a distinct part of the epiFFORMA modeling pipeline: synthetic data generation, time series pre-processing, feature extraction, component forecast modeling, GBM meta-modeling, and uncertainty quantification.

3.1. *Synthetic Data Generation.* We primarily use variations on and mixtures of the Susceptible-Infected-Recovered (SIR) Model to generate the synthetic time series data used to train epiFFORMA, to provide a library of time series segments to the method of analogues component model, and to train our Uncertainty Quantification (UQ) models. For each of these three objectives, we first generate distinct sets of 60,000 time series (20,000 per variety described below).

Both the epiFFORMA and the UQ methods require a training and testing dataset. To generate these sets, we perform the following 180000 times:

- Randomly select one of the 60,000 time series
- Randomly select a cutpoint $t$ proportional to the number of cases observed at time $t$. This strategy upweights times with relatively higher cases.

- When training epifforma, we will use observations between time 1 and $t$ to predict observations for $t+1, \ldots, t+h$, where we set $h = 4$.

Details on the three approaches used to generate the synthetic time series are provided below. All time series outputted are originally proportions between 0 and 1. After implementing the methods described below, all three sets of time series go through the following additional processing:

1. Generate a new time series by sampling from a $\mathrm{Beta}$ distribution with parameters $\alpha \cdot ts$ and $\alpha(1-ts)$, where $ts$ is the original time series and $\log(\alpha)$ is drawn from a $\mathrm{Unif}(\log(50), \log(1000))$ distribution.
2. Randomly determine whether the series represents proportions or counts. Both types of timeseries are included in the synthetic library so that the model can reasonably make forecasts on both types of data.
3. If it represents proportions, rescale the time series so that its values fall between 0 and $\pi$, where $\log(\pi)$ is sampled from a $\mathrm{Unif}(\log(0.0005), \log(0.25))$ distribution.
4. If it represents counts, scale the time series by a population size, which is the square of a sample from $\mathrm{Unif}(2 \times 10^5, 10^8)$, rounded to the nearest integer.
5. If the time series type is not "seasonal," pad both the beginning and end of the series with zeros.

*Susceptible-Infected-Recovered "Rollercoaster" Model.* This model is denoted sir-rollercoaster in the main paper. In this generative model, several Susceptible-Infected-Recovered (SIR) curves are sampled according to the process described below and combined in a way to simulate waves of infections in sequence. To construct each time series, a SIR curve is generated for each wave, where the number of waves is randomly sampled to be between 3 and 7. Randomly-selected subsets ranging from 10 to 52 timepoints of each SIR curve are combined sequentially, where either SIR curves with larger modes are more likely to occur earlier on, SIR curves with larger modes are less likely to occur earlier on, or the timing of the SIR curves is not sorted based on the SIR modes.

Each SIR curve is generated as a realization from an SIR model. The SIR Model model describes the dynamics of a disease spread by breaking a population into three set compartments and modeling the process by which individuals transition through these compartments. Denote the proportion of the population in the three compartments – susceptible, infectious, and removed – by $S_t, I_t$, and $R_t$, respectively, such that $S_t + I_t + R_t = 1$ for all $t$. Then the SIR model is determined by the equations

$$\frac{dS_t}{dt} = -\beta S_t I_t, \tag{1a}$$

$$\frac{dI_t}{dt} = \beta S_t I_t - \gamma I_t, \tag{1b}$$

$$\frac{dR_t}{dt} = \gamma I_t, \tag{1c}$$

where $\beta > 0$ is the disease transmission rate and $\gamma > 0$ is the rate of recovery. The SIR curve is determined completely by starting parameters $\beta, \gamma$, and the initial proportions in each compartment: $S_0, I_0$, and $R_0$. The quantity $\rho := \beta S_0/\gamma$, is the average number of additional infections generated by a single new infection in a completely susceptible population. An *SIR curve* is typically interpreted as the number of infected at a given time $t$: $I_t$.

The starting parameters and initial conditions for each synthetic datum from the SIR model are randomly sampled. The initial conditions are sampled from,

$$\begin{pmatrix} S_0 & I_0 & R_0 \end{pmatrix} \sim \mathrm{Dirichlet}(1000, 0.1, 0.1),$$

and the starting parameters are sampled from

$$\rho \sim 1.1 + 20X \quad \gamma \sim 1/Y,$$

where $X \sim \mathrm{Beta}(1,3)$ and $Y \sim \mathrm{Unif}(1,10)$. This sampling approach yields basic reproduction number ($\rho$) values that generally fall between 1.1 and 19.2. This span encompasses estimated $\rho$ values for a variety of diseases, including COVID-19 (10), Influenza (1), Monkeypox (6), as well as Dengue Fever, Zika, and Chikungunya (11).

*Susceptible-Infected-Recovered "Wiggle" Model.* This model is denoted sir-wiggle in the main paper. In this generative model, a component of periodicity and deviation from the standard SIR model is introduced into the SIR structure described in Section 3.1. Let $Z := Z_1, \ldots, Z_T$ denote a synthetic SIR curve generated using the procedure described above. Each time point $Z_t$ in the sequence $Z$ is then transformed according to the formula

$$Z'_t := Z_t \cdot [1 + (1 - 2A) \cdot (1 + B \cdot \sin(\pi t C))],$$

where $A \sim \mathrm{Unif}(1,2)$, $B \sim \mathrm{Binomial}(0.5)$, and $C \sim \left(\mathrm{Unif}\left(\sqrt{2/T}, \sqrt{10/T}\right)\right)^2$. The resulting synthetic sequence $Z' = Z'_1, \ldots, Z'_T$ is then included in the dataset library.

*Seasonal Model.* This model is referred to as seasonal in the main text. In this generative process, a single SIR curve is produced and repeated across multiple seasons, with a multiplicative trend applied over time. The number of seasonal repetitions is randomly selected between 5 and 15. The SIR curve is generated as previously described, except that $\rho = 1.2 + 10X$, where $X$ is drawn from a *Beta* distribution as before. Seasons follow a 52-week cycle.

The multiplicative scaling across seasons is determined by sampling: with 70% probability, it is fixed at 1; with 30% probability, it follows the form $\exp(a \cdot n)/(1 + \exp(a \cdot n))$, where $n$ indexes the number of waves and $a$ is drawn from a normal distribution with mean 0 and standard deviation 0.5. Each seasonal time series is then converted to a 4-week cadence (from its original weekly cadence) with 50% probability.

*Comment on synthetic noise and reporting delay.* Noise and bias in the observed data is represented in the synthetic data by adding in bias (to the rollercoaster trends) and noise into the synthetic data, as described above. The fact that the noise/bias can change over time in real data for the same geography/disease is addressed by the rolling window that is intrinsic to epiFFORMA. EpiFFORMA computes summaries of recent data: if noise and bias are changing over time in real data, those changes will be captured and will fall out of epiFFORMA's summary window allowing epiFFORMA to naturally adapt to changing noise/bias in the reporting. This being said, dynamics caused by reported delays are not directly simulated in the synthetic data, though this is an interest for further research.

3.2. *Time Series Pre-Processing.* Prior to smoothing, feature extraction, and component forecasting, we run time series we want to forecast through three pre-processing steps: outlier filtering, distribution of observations with zero reported cases across weeks, and time series smoothing. We found that these pre-processing steps improved the stability and reasonableness of the resulting feature estimates and forecasts. We want to emphasize that all evaluations of the proposed forecasting methods are based on the raw time series, not the pre-processed and/or smoothed time series; rather, these pre-processing steps can be viewed as internal to the forecast modeling methodology.

*Outlier Filtering.* In order to make our forecasts more robust to outliers, we performed an outlier filtering step, where outliers were replaced by fitted values. We defined outliers conservatively as follows:

- We applied the *tsoutliers* function in the R package *forecast* to identify candidates for outliers.
- Exclude any outliers that are not isolated in time
- Do not let the last observed observation be an outlier unless it is nonzero and is at least 5 times the maximum value observed in the prior 2 years of observations.
- Replace outliers with NA and interpolate their values using the *na.interp* function from the R *forecast* package.

We then apply a final filtering step that identifies isolated zeros surrounded by nonzero values and determines whether they are a likely outlier. An isolated zero is determined to be an outlier based on a generalized additive model (GAM) fit applied to the observed time series data. We implemented GAM modeling using the *mgcv* package in R. For counts time series data, we fit a Poisson distribution GAM to the observed data, and we fit a Gaussian distribution GAM to observed proportion time series data. For both types of time series, we then defined an isolated zero observation at time $k$ to be an outlier if the outputted probability of observing a zero at time $k$ from the regression fit was less than 0.05.

*Distribution of Zeros.* After outlier filtering, we considered observations in which the reported cases were exactly zero, ignoring observations occurring before the first reported case. For these observations and the two observations immediately before and after, we replace their values with values from a smoothed version of the time series, where the smoothed time series is obtained by applying a 5-observation median filter to the entire time series. This data correction step was important in settings where a single observation with zero reported cases was surrounded by observations in time with large nonzero reported cases.

*Time Series Smoothing.* Prior to feature extraction and component model forecasting, we obtained a smoothed version of the time series. This smoothed time series was used for feature extraction and forecasting for a subset of the features and component models; other features and models were based on the outlier filtered and zero-distributed time series without additional smoothing.

A GAM as implemented in the R package *mgcv* was used for time series smoothing, where the fitted values from the GAM fit provided our smoothed time series. Our GAM fit implementation used a discrete approximation of a GAM where cases were modeled using P-splines with penalization on the first and second derivatives of the resulting spline function. This GAM was fit to the last 16 observations (i.e., the last 4 months for weekly data), with the most recent observations (relative to the forecast horizon) upweighted in the model. We fit this model only to the most recent 16 observations to allow for better local estimation of the GAM smoothing parameter that controls the smoothness of the time series fit.

To help reduce the sensitivity of the GAM fits to noise in settings with case counts less than 20, we made GAM predictions in this low-counts setting on the time series after applying a 3-observation rolling median filter. In rare settings with very noisy data, the GAM fitted values were all exactly flat in time. In this case, the smoothed time series was obtained using a 4-observation rolling median filter. Time series with less than 16 unique values were not smoothed.

3.3. *Feature Extraction.* After outlier filtering, handling the distribution of zeros, and smoothing the time series, we calculated features of the smoothed or unsmoothed time series as follows. These features were constructed to facilitate discrimination between different infectious disease forecasting scenarios (i.e. when the cases are rising, when a case wave is near the peak, when cases are decreasing, etc). In defining the features, it will be helpful to recall some notation. Let $\{y_1, \ldots, y_t\}$ be the filtered time series and let $\{z_1, \ldots, z_t\}$ be the corresponding time series after GAM smoothing as described above.

*gr12_div_23.* This feature is an expression of the rate of change of the time series in the recent past. This feature's role is to capture the local time series trend. We calculated this feature as

$$tanh\left(\frac{z_t + z_{t-1}}{z_{t-1} + z_{t-2}} - 1\right).$$

*last_div_max.* This feature captures how large the last value is, relative to the minimum and maximum of the last 10 values. To stabilize this minimum and maximum estimation in the presence of high noise or remaining outliers, we calculated the minimum and maximum of the smoothed time series after applying an additional 3-observation mean filter. Let $\{v_1, \ldots, v_t\}$ denote this doubly-smoothed time series. We calculate the feature as

$$\frac{v_t - min(v_{t-9}, \ldots, v_t)}{max(v_{t-9}, \ldots, v_t) - min(v_{t-9}, \ldots, v_t)}.$$

*coefvar.* This feature is the coefficient of variation on the last 10 observations. This feature's role is to capture the local noisiness of the series. We calculate the feature as

$$tanh\left(0.1 \cdot \left\{\frac{sd([y_{t-9} - z_{t-9}], \ldots, [y_t - z_t])}{mean(z_{t-10}, \ldots, z_t)} - 1\right\}\right).$$

*gam_with_div_without.* For this feature, we obtain GAM-based forecasts for the next $h$ observations from the GAM fits used for time series smoothing. The also fit a version of the GAM that excludes $y_t$ from the fitting. The role of this feature is to capture how sensitive forecasts may be to the last time series observation. This feature is calculated as

$$tanh\left(0.1 \cdot \left\{\frac{\text{forecast using } y_t}{\text{forecast not using } y_t} - 1\right\}\right).$$

*avg_recent_div_avg_global.* Let $m$ be the index of observation occurring two years prior to time $t$. This feature captures the magnitude of cases in the last 10 observations relative to the magnitude of cases in the last 2 years. We calculate this feature as

$$tanh\left(\frac{mean(z_{t-10}, \ldots, z_t)}{mean(z_{t-m}, \ldots, z_{t-1})} - 1\right).$$

*diff_zscore.* This feature captures the magnitude of the jump between the last two values relative to the values observed over the last two years. This feature aims to help epifforma discrimimate between final observations that are the start of a new case wave versus an outlier. We calculate this feature as

$$tanh\left(0.1 \cdot \left\{\frac{z_t - z_{t-1} - mean([z_{m+1} - z_m], \ldots, [z_t - z_{t-1}])}{sd([z_{m+1} - z_m], \ldots, [z_t - z_{t-1}])}\right\}\right).$$

*entropy.* This feature is the spectral entropy as implemented by the R package *tsfeatures* (9).

*relative_increases.* This feature captures how many consecutive increases in cases we have observed in the recent past and how this compares to the maximum number of consecutive increases observed in the past. This feature may help identify case wave peaks in settings with highly-regular seasonality. To calculate this feature, we identify all windows of increasing cases observed for the time series and their corresponding lengths, and then we calculate the proportion of the increasing intervals with lengths less that the observed number of consecutive increases at the tail of the series. In settings where there are no consecutive increases at the tail of the series, this feature takes value 0.

*prop_since_peak.* For this feature, we calculate the day/week/month that is associated with the largest value of cases on average in historical seasons. This feature is the proportion of a year since that day/week/month. This feature also helps to identifying where in the season we are forecasting and may be helpful for forecasting highly-seasonal diseases.

*seasonality.* This feature aims to capture the degree of regularly-timed seasonality in the time series. We clarify that we are interested in capturing seasonality in which the seasonal case waves have very predictable timing and peak locations. We calculate this feature as the maximum autocorrelation of the time series, considering possible lags between 0.5 and 1.5 times the number of observations per year. This feature is calculated after excluding leading zeros at the start of the time series.

3.4. *Component Forecast Modeling.* All of the following component forecasting models were applied to the time series or a smoothed version of the time series up to time $t$ and used to forecast for time intervals $t+1, ..., t+h$. All results presented in the main paper use $h = 4$.

As with time series feature extraction, the component modeling is implemented on time series that have already passed through an outlier filter and possible zero re-distribution as described in **Supp. Section 3.2**.

Some component models were applied to the time series itself (gam, moa, moa_deriv, theta, meanfcst) and other were applied to a smoothed version of the time series (rw, mirror, gam2mirror, arima). The role of smoothing was to stabilize the corresponding component model forecasts in the presence of high noise in the inputted time series.

*Generalized Additive Model (GAM).* This model is denoted gam in the main paper. This is a generalized additive model (GAM) as implemented in the R package *mgcv*. This component model used for forecasting is the same used to generate smoothed time series as described above.

In rare settings with very noisy data, the GAM fitted values and predictions were all exactly flat in time. In this case, forecasts were obtained using a 4-observation rolling median filter and carrying the final observation forward. For time series with less than 16 unique values, forecasts were obtained by carrying the last observation forward without any additional smoothing or filtering.

*Random walk forecast.* This model is denoted rw in the main paper and corresponds to a simple random walk model without any drift as implemented in the R package *forecast*.

*Theta Model.* This model is denoted theta in the main paper and corresponds to simple exponential smoothing with drift as implemented in the R package *forecast*.

*Arima Model.* This model is denoted arima in the main paper and corresponds to a autoregressive integrated moving average model. This model depends on hyperparameters related to the temporal dependence structure, and these were estimated using the AICc criterion and other default settings in the *auto.arima* function in the R package *forecast*.

*Method of Analogues.* This model is denoted moa in the main paper, and is developed in (16). The method of analogues historically works by building a library of historical time series observation chunks and defining the forecast based on the historical time series chunks closest to the observed values we want to forecast.

In our implementation, we define the library used for forecasting based on *simulated* time series data instead of historical data. This allows the library to be much larger and to cover a wider space of disease dynamics. The synthetic library used for our moa component model is separate than the one used to fit the epiFFORMA GBM, yet was generated using the same methods applied to generate the synthetic datasets used to train epiFFORMA and used for uncertainty quantification. For a given time series, the component model forecasts are obtained as a median of the 2000 nearest simulated time series chunks, where nearness was defined using a simple pointwise absolute error distance metric based on the most recent 4 observations.

A detailed exploration of the performance of a synthetic method of analogues can be found in (16).

*Method of Anaologues on Derivatives/Differences.* This model is denoted moa_deriv in the main paper, and is developed in (16). This is a synthetic data-based method of analogues as described above, with an additional modification. Instead of defining the nearest time series library members based on the observed time series, nearness is evaluated based on the *differences* between sequential observations. Observed time series differences are matched to library chunk differences again using a simple sum of squared errors distance metric applied to the most recent 5 observations. Unlike the moa component model, the moa_deriv is less sensitive to the magnitude of the case time series; instead, it only relies on matching differences/derivatives.

*Mean Forecast.* This model is denoted meanfcst in the main paper and is the mean of the observed cases over the least two years of data. This component model is restricted to the last two years to better handle drift in seasonal diseases.

*Mirror Model.* This model is denoted mirror in the main paper. This model defines the forecast for time $t+k$ as the observed value for time $t-k$. Forecasts for $k$ observations after time $t$ would simply be the observations at $t-1, \ldots, t-k$. The goal of this odd forecast model formulation was to provide a high-quality forecast at the exact time of a case wave peak; this model is expected to perform poorly for most times. For this reason, the mirror model was excluded from the equal-weights ensemble calculation.

*GAM to Mirror Model.* This model is denoted gam2mirror in the main paper. The gam component model extrapolates linearly, and this model serves to generalize the gam forecast to allow for turn-overs near the peak of a case wave. This component defines a sequence of $h$ decreasing values between 0.9 and 0.1 as weights and assigns the forecast as as weighted combination of the gam and mirror forecasts, where higher weights are placed on the gam values for nearer-term forecasts and higher weights are placed on the mirror values for longer-term forecasts.

3.5. *GBM Meta-Model.* Following recommendations by (15), we estimated ensemble weights using a GBM meta-model. A GBM is used due to its fast prediction time, its ability to handle multiple features of varying data types, and because it only requires the optimization of a relatively small number of parameters. This method was used by every contestant ranked in the top 50 in the M5 forecasting competition, and has become a standard for several

other forecasting applications (see (12) for more details on the prevalence of `lightGBM`).

One option for training this GBM would be to define class labels associated with the $K$ different component forecasting models. Interestingly, we found improved performance by defining the labels based on the *ordering* of these component models; i.e., with class 1 denoting the smallest forecast and class $K$ denoting the largest forecast. Tied forecast pairs were randomly ordered. We hypothesize that this approach allowed the GBM to indirectly serve as a regressor in addition to a classifier, where the GBM can encode preferences toward higher or lower forecasts as a function of time series features into corresponding forecast weights.

During the course of model development, we considered several different strategies for training this GBM classifier based on the synthetic training dataset. We ultimately chose to construct a GBM training dataset with multiple rows per training data + split combination, where multiple rows corresponded to different component forecast models. Quality-of-forecast weights were assigned to each of these rows, where the weights were inversely proportional to the squared error between the component model forecast and the observed data. To reduce the size of the training data (useful to speed up computation), we excluded all training data point + component model combination with corresponding quality-of-forecast weight less than 0.99/K, where $K$ is the number of component forecast models.

We also had some choice in the loss function used to train the GBM model. We compared performance under two different loss functions: a mean squared error-based loss function and a multinomial log-likelihood-based loss function. We observed slightly better performance under the mean squared error-based loss function, and all results presented in the main paper and supplement use this loss function.

The GBM fitting depends on a variety of tuning parameters, specifically the parameters num_leaves, learning_rate, feature_fraction, max_depth, early_stopping_rounds, and prop_holdout (see (4) for details). To address the challenge of specifying these tuning parameters, we implemented a Bayesian optimization approach using synthetic data as in (16). In particular, we define an optimization function that calculates the cross-validated logloss of the `lightGBM` model on a set of synthetic data. We set the Bayesian Optimization scheme to determine the appropriate set of tuning of parameters that minimize this logloss using the process in (20).

The final GBM ensemble weights used to obtain the final epifforma forecasts were calculated as the mean of 7 sets of weights from 7 GBM fits. This GBM ensembling step served to stabilize the estimated weights across multiple bagging training data splits. We chose to use 7 GBM fits simply due to convenience based on availability of computational resources.

3.6. *Equal Weights Ensemble.* A natural competitor to the GBM weighted ensemble is a simple equal-weighted ensemble of the GBM component models. In our evaluation of our GBM ensemble, therefore, an equal-weighted ensemble forecaster will be used to benchmark performance. In calculating these equal-weighted forecasts, we exclude the mirror component model from consideration. The forecast for time $t + k$ under this equal-weighted model is therefore just a simple average for the $t + k$ forecasts for all component models, excluding the mirror model.

3.7. *Uncertainty Quantification.* For uncertainty quantification (UQ), we compared a variety of methods, but here we present only the best-performing method. This method can be viewed as an epiFFORMA-type ensemble method applied to the widths of predictive intervals as follows. Using a separate set of synthetic data generated as described in **Supp. Section 3**, construct a time series + split dataset for training the UQ models. For each time series + split in the training data, calculate the epifforma model predictions for the next $h$ observations.

For each time series + split, we also calculate three varieties of prediction interval widths for the next $h$ observations as follows:

1. Fit one of the gam, arima, or theta component forecasting models used within epifforma as described previously.
2. For the gam forecasting model fit, 95% *confidence* intervals for the expected future observation can be outputted from the software easily, but additional work must be done to calculate corresponding *prediction intervals* (which also incorporate noise in the observed values around their expectations). One strategy to obtain prediction intervals would be to add $2 * 1.96 * \sigma$ to the confidence interval widths, where $\sigma$ is the observation error estimated as part of the gam fit. However, this approach assumes homoscedasticity of the errors; that is, that the distribution of the difference between the observed data and the mean model has a constant variance. Violations of this assumption are evident in simulated and real data. To address the heterscedasticity evident in the data, we instead obtain prediction intervals by adding $2 * 1.96 * \sigma_{new}(h)$, where $\sigma_{new}(h)$ is estimated by: (a) calculating the absolute difference between the fitted and observed values for observations in the current training time series prior to the split, (b) modeling the absolute difference using another gam "error" model fit as a function of the observed values, and (c) evaluating the error model at the $h$ time series forecasts from the forecasting model fit, we obtain $\sigma_{new}(h)$. These are a function of $h$ and provide a much improved estimate of the prediction error in the presence of heteroscedasticity relative to the standard software outputs.

3. For the arima and theta forecasting models, 95% prediction intervals are readily available using the *forecast* package in *R*. However, these prediction intervals also suffer from poor performance related to homoscedasticity assumptions made in the forecasting modeling. To address this challenge, we use the same approach as above to estimate $\sigma_{new}(h)$, this time modeling the absolute difference between the arima or theta fitted values and the time series observations. We can obtain our heteroscedasticity-adaptable prediction interval widths the 95% confidence interval widths + $2 * 1.96 * \sigma_{new}(h)$, where the 95% confidence interval widths are obtained by subtracting $2 * 1.96 * \sigma$ from the standard prediction interval widths obtained from the *forecast* package in *R*.

Given a time series, split, epifforma predictions, and three sets of component prediction interval widths (denoted $W_{[k]}(h,t)$), we calculate the final epifforma prediction interval width as follows:

(2)
$$\hat{W}(h,t) = \sum_{k=1}^{3} \alpha_{[k]} \cdot \hat{W}_{[k]}(h,t),$$

where $\alpha_{[k]}$ is a set of weights estimated using a GBM much like the GBM used to train epifforma forecast model weights. Unlike the main epifforma GBM, this UQ GBM is trained with three outcome classes (one for each of the three prediction interval widths).

In order to train this UQ GBM, we calculate the three component prediction intervals for every time series + split in the UQ training dataset. For each time series + split, we assign a weight associated with the quality of the corresponding predictive interval. These training data weights are inversely proportional to the interval score associated with each prediction interval, where the interval score is defined as follows:

$$\hat{U}(h,t) - \hat{L}(h,t) + \frac{2}{0.05}(\hat{L}(h,t) - y_{t+h})I(y_{t+h} < \hat{L}(h,t))$$
$$+ \frac{2}{0.05}(y_{t+h} - \hat{U}(h,t))I(y_{t+h} > \hat{U}(h,t)),$$

where $\hat{Y}(h,t)$ is the epifforma forecast, where $\hat{L}(h,t) = max(0, \hat{Y}(h,t) - 0.5 * \hat{W}_{[k]}(h,t))$ is the lower bound of the 95% prediction interval, and where $\hat{U}(h,t) = max(0, \hat{Y}(h,t) + 0.5 * \hat{W}_{[k]}(h,t))$ is the upper bound of the 95% prediction interval (2). These prediction intervals are assumed to be symmetric around the epifforma forecast (except when truncated below by zero).

**4. Data Sources and Cleaning.** All data sources used in this manuscript were publicly-available. Below, we detail access information for these data sources and summarize the data processing choices such as exclusion criteria. On all data, we evaluate on the unsmoothed time series with outliers and extra zeros.

4.1. *COVID-19.* Data on new confirmed COVID-19 cases by location were sourced from the Johns Hopkins University Center for Systems Science and Engineering GitHub repository available at https://github.com/CSSEGISandData/COVID-19_Unified-Dataset on September 20th, 2023. These data consisted of cases reported between January 2nd, 2020 and March 31st, 2023. Daily data were aggregated up to the weekly scale to avoid day of week effects, where weeks were defined using the US CDC version of epidemiological weeks as implemented in the R package *lubridate*.

We further subsetted the data by excluding data from locations with 10 or less weeks with non-zero reported cases and/or with less than 1000 cumulative cases ever reported. For our global COVID-19 analysis, this resulted in a total of 193 location time series. Zeros at the start and end of each time series were also truncated off, since those times are relatively uninteresting for forecasting. Rare negative reported case values were set to zero.

An additional data processing step was applied to the latter portion of the case time series reported for Florida. These data exhibited an odd periodic oscillation in reporting spread over weeks. For these weeks, we defined the “true" case data based on a 4-week rolling average applied to the latter half of the time series, where the cases assigned to week t used data collected between weeks t-3 to week t.

4.2. *ILI.* Weekly data on new influenza-like illness (ILI) diagnoses in the United States were downloaded from the US CDC at https://gis.cdc.gov/grasp/fluview/fluportaldashboard.html on or before January 31st, 2024. Data aggregated by census regions, health and human services (HHS) regions, and national data were also included as separate locations for forecasting. ILI in these data was defined as the proportion of recorded outpatient visits associated with an ILI diagnosis, resulting in a time series of proportions for each location. These represented ILI diagnoses occurring between 2010 and 2024 at the US state level and between 1997 and 2024 for larger spatial regions.

4.3. *Dengue Fever.* Weekly dengue fever data was obtained from several different sources. Firstly, data for cases reported in Iquitos, Peru (2000-2009) and San Juan, Puerto Rico (1990-2009) were obtained from https://dengueforecasting.noaa.gov. Weekly case time series for other locations were obtained from https://opendengue.org on May 14, 2024 (3). Data collected prior to 1990 were excluded, as were locations with less than 52 total weeks of data or otherwise sparsely reported data. Our final dataset included cases reported for Vietnam, The United States, Sri Lanka, Singapore, Paraguay, Peru (Overall), Peru (Iquitos), Panama, Puerto Rico (San Juan), Nicaragua, Mexico, Malaysia, Lao People's Democratic Republic, Honduras, El Salvador, Ecuador, Dominican Republic, Costa Rica, Colombia, Cambodia, Brazil, Bolivia, Belize, Barbados, and Argentina.

4.4. *Chikungunya (Brazil).* Weekly data on laboratory-confirmed Chikungunya cases in Brazil (by state) from 2013 to 2022 were obtained at https://github.com/wmarciel/Chikungunya-in-Brazil-2013-2022. These data were originally sourced from the Brazilian Ministry of Health by (5).

4.5. *Diphtheria, Measles, Mumps, Polio, Rubella, and Smallpox.* Weekly data for these diseases were obtained from Project Tycho at https://www.tycho.pitt.edu/data/#datasets (19). Data were aggregated up to a 4-week cadence. Excess zeros at the start and end of each location's time series were truncated, and locations with less than 5000 reported cases or reported cases for 10 or less 4-week periods for a given disease were excluded from analysis.

**5. Assessment of Synthetic Data Coverage.** As stated in the main paper, the synthetic data library was designed with the goal of broadly covering many disease dynamics while also not being trained to resemble any one disease. Here, we evaluate how well the synthetic data effectively "cover" the real-world data described in Table 1 in the main paper.

It's important to note that this analysis was conducted post hoc, after generating the synthetic data using the methods described previously. The results here were not used to refine or adjust the synthetic dataset to better align with any particular disease. While enhancing coverage might improve epiFFORMA's performance, such adjustments would diverge from the primary aim of this study: to evaluate how well a fixed, broadly generated synthetic dataset performs for previously unseen diseases. The current results—along with those in the main paper—illustrate the surprising utility of the deliberately *ad hoc* synthetic data generation process from Section 3. That said, future work will explore ways to enrich the synthetic data library, potentially via agent-based modeling or experiments where disease-specific data are selectively included or excluded.

5.1. *Dimensionality Reduction via Uniform Manifold Approximation and Projection (UMAP).* Given the challenge of visualizing millions of synthetic and real timeseries, we turn to Uniform Manifold Approximation and Projection (UMAP), a method described in (8). UMAP works by modeling local relationships between high-dimensional data points as a graph and then projecting that structure into a lower-dimensional space—here, two dimensions—while preserving local similarity. In this reduced space, points that appear close together typically reflect closeness in the original high-dimensional space. For a more detailed introduction, see (14).

As an initial processing step to this analysis, we first transform each timeseries into the 10-dimensional vector of features described in Section 3.3. The aim of the following analysis is to see how well the synthetic data cover the real data in terms of this 10-dimensional feature space.

Figures S3 & S4 compare the synthetic library to real data of COVID-19, Measles, Mumps, ILI, Polio, Rubella, Smallpox, Diphtheria, and Chikungunya. The plots suggest that synthetic data align well with the real data used in the main paper in terms of the features from Section 3.3. Note that each figure represents a unique topological mapping, so while the synthetic data used in each figure are the same, they may show up in different locations.

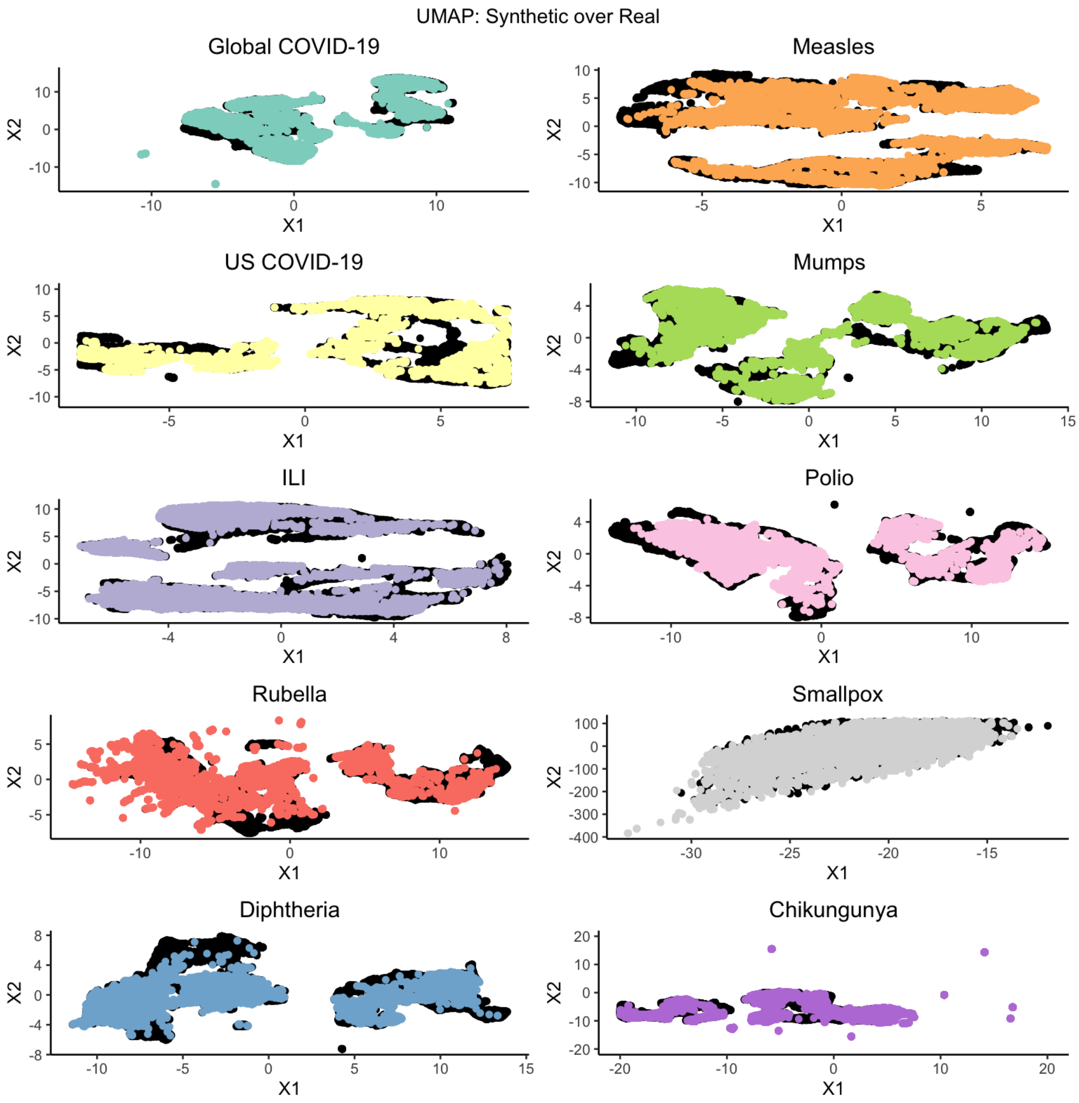


FIG S3. *Visualization of synthetic versus real data using UMAP, where the real data points are plotted on top of the synthetic data points. Black points denote synthetic data segments, while colored points represent real data from individual diseases.*

**6. Analysis of epiFFORMA by Regimes.** In the following section, we use the shapelet-based methods from (18) to develop a better intuition for the conditions under which epiFFORMA does and does not perform well. In (18), an automated process is developed that classifies an observed time series into different “regimes." These regimes are *Surge* (an initial uptick of cases), *Stable Increase* (linear increase in the number of cases after the surge and before the peak), *Near Peak* (the period prior to the peak where the increasing begins to slow), *Past Peak* (the initial decrease of cases), and Stable Decrease (linear decrease pas the peak) and *Flat* (no further increases or decreases). Splitting the data into these six types of behaviors in a disease outbreak allows us to analyze what behaviors epiFFORMA captures well and what behaviors it fails to capture.

We visualize the performance of epiFFORMA compared to the equal-weights ensemble and the individual component models in Figure S5. The boxplots summarize the averaged error metric for all geographies and forecast horizons for a given disease and regime. For

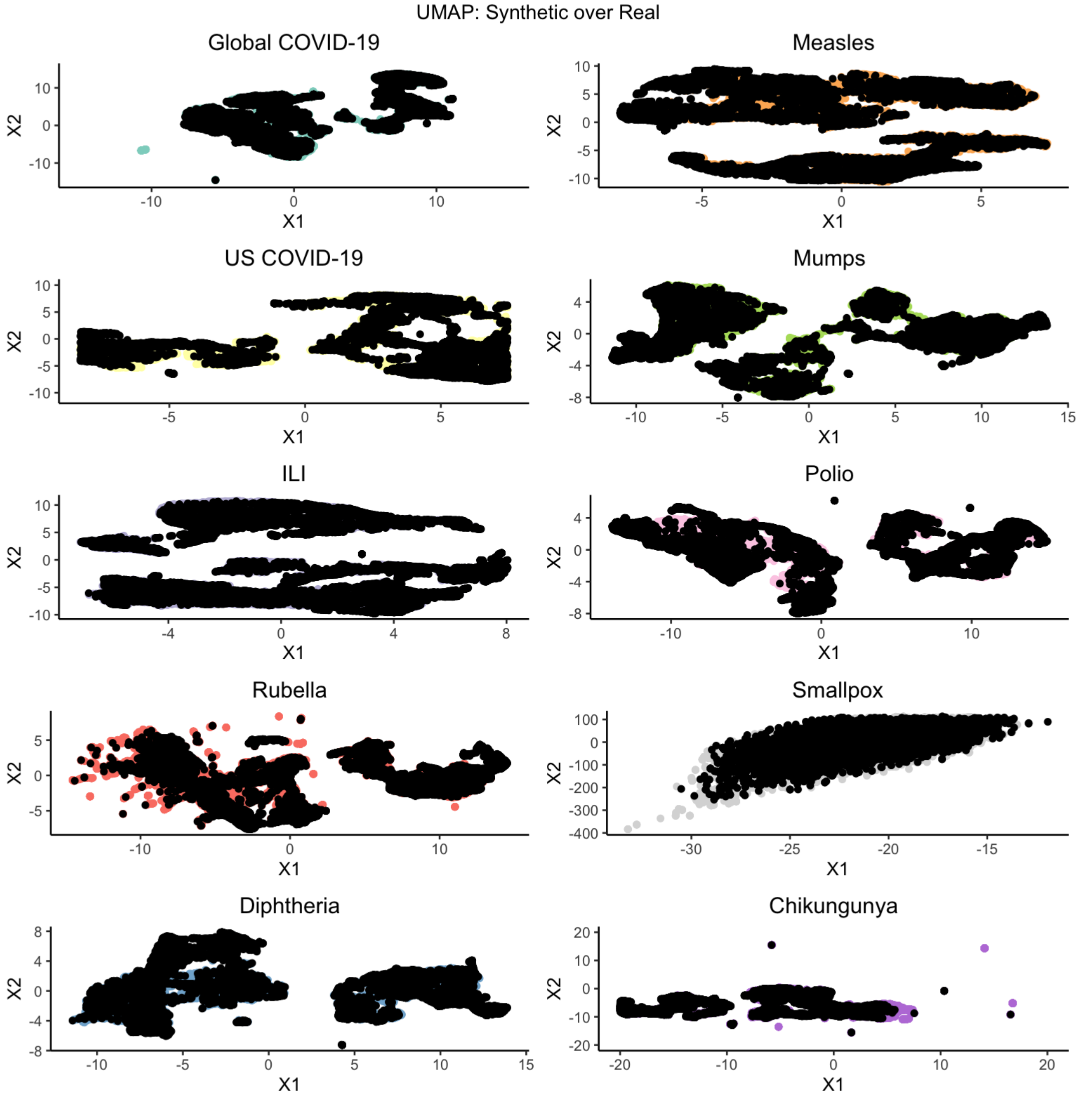


FIG S4. *Visualization of synthetic versus real data using UMAP, where the synthetic data points are plotted on top of the real data points. Black points denote synthetic data segments, while colored points represent real data from individual diseases.*

instance, every point in the top left-hand cell is an average MAE for a disease and model, averaged across geography and forecasts, only considering forecasts for segments of the time-series determined to be in a “surge" regime according to the method of (18).

Figure S5 shows that the performance between epiFFORMA and the equal-weights ensemble is mostly comparable for the Stable Increase and Near Peak regimes, that epiFFORMA has slightly better performance for the Surge and Past Peak regimes, and that epiFFORMA has significantly better performance for the Stable Decrease and Flat regimes. These results imply that at its worst, epiFFORMA reverts to an equal-weight-like scheme.

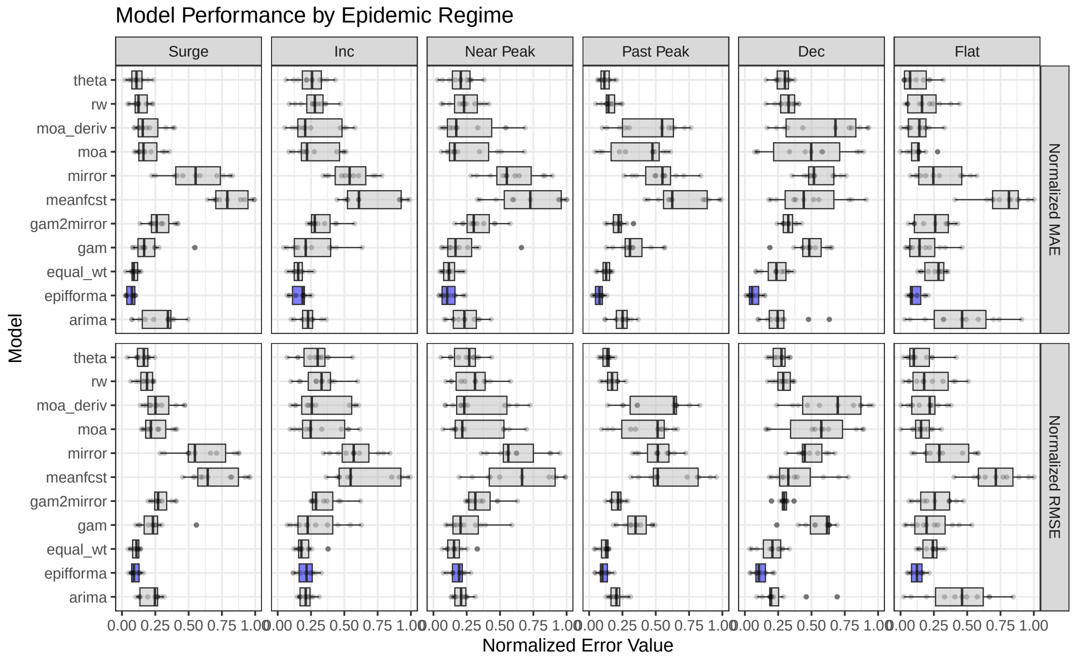


FIG S5. *Error metrics for epiFFORMA, the equal-weights ensemble (excluding the mirror model), and the individual component models, for every disease discussed in the main paper ($n = 11$), divided according to six regimes according to the methods in (18).*

**7. Comparison of epiFFORMA to alternative online ensembling techniques.** The modern literature for adaptive, online weighting strategies that do not incorporate historical data is somewhat bare. The closest that the authors found was (13), but this approach requires each component model to give probabilistic forecasts. We propose several alternative online ensembling strategies that do not incorporate historical data. These methods are as follows:

1. `kalman`: a Kalman Filter on time-varying weights.
2. `rls`: Recursive Least Squares with forgetting factor $\lambda$ (7),
3. `ridge`: Recursive Least Squares with no forgetting factor and a ridge regression shinkage factor,
4. `roll`: At each t, regress on forecasts from last history of 40 points,
5. `equal_wt`: An equal-weights ensemble.

All of these techniques are fit to the order of models rather than to the models themselves.

We have visualized the cumulative average MAE of each of these strategies for forecasting COVID-19 in California in Figure S6. Over the course of the pandemic, the epiFFORMA technique reaches a lower average MAE than equal weights and the several online ensembling techniques considered. This figure supports the observations made in (17), and continues to show that epiFFORMA does better than equal weights.

## REFERENCES

[1] BIGGERSTAFF, M., CAUCHEMEZ, S., REED, C., GAMBHIR, M. and FINELLI, L. (2014). Estimates of the reproduction number for seasonal, pandemic, and zoonotic influenza: a systematic review of the literature. *BMC Infectious Diseases* **14** 480. https://doi.org/10.1186/1471-2334-14-480

[2] BRACHER, J., RAY, E. L., GNEITING, T. and REICH, N. G. (2021). Evaluating epidemic forecasts in an interval format. *PLOS Computational Biology* **17** 1-15. https://doi.org/10.1371/journal.pcbi.1008618

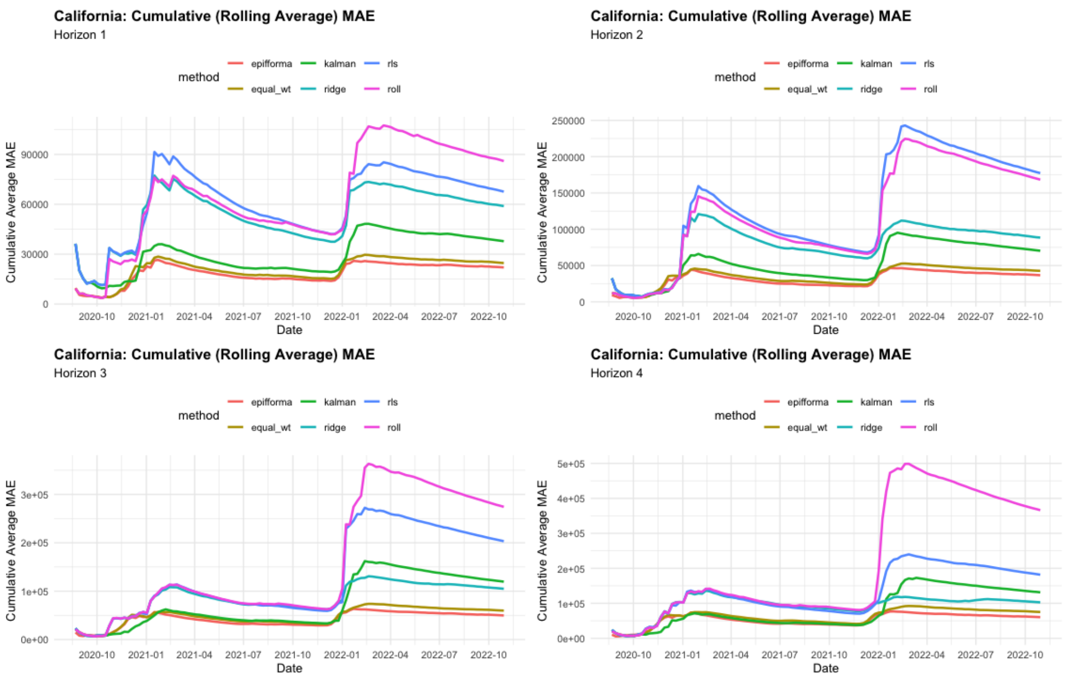


FIG S6. *Comparison of alternative ensembling techniques.*


[3] CLARKE, J., LIM, A., GUPTE, P., PIGOTT, D. M., VAN PANHUIS, W. G. and BRADY, O. J. (2024). A global dataset of publicly available dengue case count data. *Scientific Data* **11** 1-14. https://doi.org/10.1038/s41597-024-03120-7

[4] THE MICROSOFT CORPORATION (2023). LightGBM Documentation. https://lightgbm.readthedocs.io/en/stable/index.html.

[5] DE SOUZA, W. M., DE LIMA, S. T. S., SIMÕES MELLO, L. M., CANDIDO, D. S., BUSS, L., WHITTAKER, C., CLARO, I. M., CHANDRADEVA, N., GRANJA, F., DE JESUS, R., LEMOS, P. S., TOLEDO-TEIXEIRA, D. A., BARBOSA, P. P., FIRMINO, A. C. L., AMORIM, M. R., DUARTE, L. M. F., PESSOA, I. B. J., FORATO, J., VASCONCELOS, I. L., MAXIMO, A. C. B. M., ARAÚJO, E. L. L., PERDIGÃO MELLO, L., SABINO, E. C., PROENÇA-MÓDENA, J., FARIA, N. R. and WEAVER, S. C. (2023). Spatiotemporal dynamics and recurrence of chikungunya virus in Brazil: an epidemiological study. *Lancet Microbe* **4** e319-e329. https://doi.org/10.1016/S2666-5247(23)00033-2

[6] DÍAZ-BROCHERO, C. and CUCUNUBÁ, Z. M. (2024). Epidemiological findings, estimates of the instantaneous reproduction number, and control strategies of the first Mpox outbreak in Latin America. *Travel Medicine and Infectious Disease* **59** 102701. https://doi.org/10.1016/j.tmaid.2024.102701

[7] FARHANG-BOROUJENY, B. (2013). *Adaptive Filters: Theory and Applications*, 2nd ed. John Wiley & Sons.

[8] HEALY, J. and MCINNES, L. (2024). Uniform manifold approximation and projection. *Nature Reviews Methods Primers* **4** 82. https://doi.org/10.1038/s43586-024-00363-x

[9] HYNDMAN, R., KANG, Y., MONTERO-MANSO, P., O'HARA-WILD, M., TALAGALA, T., WANG, E. and YANG, Y. (2023). tsfeatures: Time Series Feature Extraction R package version 1.1.1.

[10] LINKA, K., PEIRLINCK, M. and KUHL, E. (2020). The reproduction number of COVID-19 and its correlation with public health interventions. *medRxiv*. https://doi.org/10.1101/2020.05.01.20088047

[11] LIU, Y., LILLEPOLD, K., SEMENZA, J. C., TOZAN, Y., QUAM, M. B. M. and ROCKLÖV, J. (2020). Reviewing estimates of the basic reproduction number for dengue, Zika and chikungunya across global climate zones. *Environmental Research* **182** 109114. https://doi.org/10.1016/j.envres.2020.109114

[12] MAKRIDAKIS, S., SPILIOTIS, E. and ASSIMAKOPOULOS, V. (2022). M5 accuracy competition: Results, findings, and conclusions. *International Journal of Forecasting* **38** 1346-1364. Special Issue: M5 competition. https://doi.org/10.1016/j.ijforecast.2021.11.013

[13] MCANDREW, T. and REICH, N. G. (2021). Adaptively stacking ensembles for influenza forecasting. *Statistics in Medicine* **40** 6931-6952. https://doi.org/10.1002/sim.9219

[14] McInnes, L. (2018). How UMAP Works. Accessed on January 9, 2025.

[15] Montero-Manso, P., Athanasopoulos, G., Hyndman, R. J. and Talagala, T. S. (2020). FFORMA: Feature-based forecast model averaging. *International Journal of Forecasting* **36** 86–92.

[16] Murph, A. C., Gibson, G. C., Amona, E. B., Beesley, L. J., Castro, L. A., Del Valle, S. Y. and Osthus, D. (2025). Synthetic method of analogues for emerging infectious disease forecasting. *PLOS Computational Biology* **21** 1-17. https://doi.org/10.1371/journal.pcbi.1013203

[17] Ray, E. L., Brooks, L. C., Bien, J., Biggerstaff, M., Bosse, N. I., Bracher, J., Cramer, E. Y., Funk, S., Gerding, A., Johansson, M. A., Rumack, A., Wang, Y., Zorn, M., Tibshirani, R. J. and Reich, N. G. (2023). Comparing trained and untrained probabilistic ensemble forecasts of COVID-19 cases and deaths in the United States. *International Journal of Forecasting* **39** 1366-1383. https://doi.org/10.1016/j.ijforecast.2022.06.005

[18] Srivastava, A., Singh, S. and Lee, F. (2022). Shape-based Evaluation of Epidemic Forecasts. In *2022 IEEE International Conference on Big Data (Big Data)* 1701-1710. https://doi.org/10.1109/BigData55660.2022.10020895

[19] van Panhuis, W. G., Cross, A. and Burke, D. S. (2018). Project Tycho 2.0: a repository to improve the integration and reuse of data for global population health. *Journal of the American Medical Informatics Association* **25** 1608-1617. https://doi.org/10.1093/jamia/ocy123

[20] Wilson, S. (2022). ParBayesianOptimization: Parallel Bayesian Optimization of Hyperparameters R package version 1.2.6.
