# Supplementary material for: A disease-agnostic approach to ensemble learning for infectious disease forecasting
Source: Nat Commun. 2026 Mar 20;17:4255. doi: 10.1038/s41467-026-70937-8 (PMC13168361; doi:10.1038/s41467-026-70937-8)
Supplement: Supplementary file 2 — Reporting Summary [file 41467_2026_70937_MOESM2_ESM.pdf]

nature portfolio

Corresponding author(s): AC Murph and LJ Beesley

Last updated by author(s): 2025/12/24

Reporting Summary

Nature Portfolio wishes to improve the reproducibility of the work that we publish. This form provides structure for consistency and transparency in reporting. For further information on Nature Portfolio policies, see our Editorial Policies and the Editorial Policy Checklist.

Statistics

For all statistical analyses, confirm that the following items are present in the figure legend, table legend, main text, or Methods section.

n/a	Confirmed	
☐	☒	The exact sample size (n) for each experimental group/condition, given as a discrete number and unit of measurement
☐	☒	A statement on whether measurements were taken from distinct samples or whether the same sample was measured repeatedly
☒	☐	The statistical test(s) used AND whether they are one- or two-sided<br>*Only common tests should be described solely by name; describe more complex techniques in the Methods section.*
☐	☒	A description of all covariates tested
☐	☒	A description of any assumptions or corrections, such as tests of normality and adjustment for multiple comparisons
☐	☒	A full description of the statistical parameters including central tendency (e.g. means) or other basic estimates (e.g. regression coefficient) AND variation (e.g. standard deviation) or associated estimates of uncertainty (e.g. confidence intervals)
☒	☐	For null hypothesis testing, the test statistic (e.g. F, t, r) with confidence intervals, effect sizes, degrees of freedom and P value noted<br>*Give P values as exact values whenever suitable.*
☒	☐	For Bayesian analysis, information on the choice of priors and Markov chain Monte Carlo settings
☒	☐	For hierarchical and complex designs, identification of the appropriate level for tests and full reporting of outcomes
☒	☐	Estimates of effect sizes (e.g. Cohen's d, Pearson's r), indicating how they were calculated

Our web collection on statistics for biologists contains articles on many of the points above.

Software and code

Policy information about availability of computer code

Data collection	Codes are available on paper's Zenodo and Github pages, as is the data. See: https://zenodo.org/records/16582447 https://github.com/lanl/precog
Data analysis	The analysis of the data and coding of the model were both done in R. See: https://zenodo.org/records/16582447 and https://github.com/lanl/precog

For manuscripts utilizing custom algorithms or software that are central to the research but not yet described in published literature, software must be made available to editors and reviewers. We strongly encourage code deposition in a community repository (e.g. GitHub). See the Nature Portfolio guidelines for submitting code & software for further information.

Data

Policy information about availability of data

All manuscripts must include a data availability statement. This statement should provide the following information, where applicable:

- Accession codes, unique identifiers, or web links for publicly available datasets
- A description of any restrictions on data availability
- For clinical datasets or third party data, please ensure that the statement adheres to our policy

All data used in this study were publicly available at the time of access.

COVID-19 case data were obtained from the Johns Hopkins University Center for Systems Science and Engineering GitHub repository (https://github.com/CSSEGISandData/COVID-19_Unified-Dataset
), accessed September 20, 2023, and include reported cases between January 2, 2020 and March 31, 2023.

Influenza-like illness (ILI) data were downloaded from the U.S. Centers for Disease Control and Prevention FluView portal (https://gis.cdc.gov/grasp/fluview/fluportaldashboard.html
), accessed on or before January 31, 2024.

Dengue fever data were obtained from the NOAA Dengue Forecasting Project (https://dengueforecasting.noaa.gov
) and OpenDengue (https://opendengue.org
), accessed May 14, 2024.

Chikungunya case data for Brazil (2013–2022) were obtained from https://github.com/wmarciel/Chikungunya-in-Brazil-2013-2022
, originally sourced from the Brazilian Ministry of Health.

Diphtheria, measles, mumps, polio, rubella, and smallpox data were obtained from Project Tycho (https://www.tycho.pitt.edu/data/#datasets
).

All data were processed as described in the Methods section, including aggregation to weekly or four-week cadences where applicable, exclusion of locations with insufficient reporting, truncation of leading and trailing excess zeros, and treatment of rare negative case counts. No new datasets were generated during the current study.

Research involving human participants, their data, or biological material

Policy information about studies with human participants or human data. See also policy information about sex, gender (identity/presentation), and sexual orientation and race, ethnicity and racism.

Reporting on sex and gender	This study did not include human test subjects, just publicly reported data on disease spread.
Reporting on race, ethnicity, or other socially relevant groupings	This study did not include human test subjects, just publicly reported data on disease spread.
Population characteristics	This study did not include human test subjects, just publicly reported data on disease spread.
Recruitment	This study did not include human test subjects, just publicly reported data on disease spread.
Ethics oversight	This study did not include human test subjects, just publicly reported data on disease spread.

Note that full information on the approval of the study protocol must also be provided in the manuscript.

Field-specific reporting

Please select the one below that is the best fit for your research. If you are not sure, read the appropriate sections before making your selection.

☐ Life sciences ☐ Behavioural & social sciences ☒ Ecological, evolutionary & environmental sciences

For a reference copy of the document with all sections, see nature.com/documents/nr-reporting-summary-flat.pdf

Ecological, evolutionary & environmental sciences study design

All studies must disclose on these points even when the disclosure is negative.

Study description	A ensembling strategy is developed using only synthetic data. This strategy is tested on publicly-available disease spread data.
Research sample	Data were not collected as part of this study.
Sampling strategy	Data were not collected as part of this study.
Data collection	COVID-19 case data were obtained from the Johns Hopkins University Center for Systems Science and Engineering GitHub repository (https://github.com/CSSEGISandData/COVID-19_Unified-Dataset<br>), accessed September 20, 2023, and include reported cases between January 2, 2020 and March 31, 2023.<br><br>Influenza-like illness (ILI) data were downloaded from the U.S. Centers for Disease Control and Prevention FluView portal (https://gis.cdc.gov/grasp/fluview/fluportaldashboard.html<br>), accessed on or before January 31, 2024.<br><br>Dengue fever data were obtained from the NOAA Dengue Forecasting Project (https://dengueforecasting.noaa.gov

) and OpenDengue (https://opendengue.org
), accessed May 14, 2024.

Chikungunya case data for Brazil (2013–2022) were obtained from https://github.com/wmarciel/Chikungunya-in-Brazil-2013-2022
, originally sourced from the Brazilian Ministry of Health.

Diphtheria, measles, mumps, polio, rubella, and smallpox data were obtained from Project Tycho (https://www.tycho.pitt.edu/data/
#datasets
).

All data were processed as described in the Methods section, including aggregation to weekly or four-week cadences where applicable, exclusion of locations with insufficient reporting, truncation of leading and trailing excess zeros, and treatment of rare negative case counts. No new datasets were generated during the current study.

Timing and spatial scale	Data were not collected as part of this study.
Data exclusions	Data were excluded only in instances where there were insufficient reporting in specific locations. More details on this are available in the text.
Reproducibility	Code should be runable from a fresh pull from Github.
Randomization	There were no experiments for which randomization of participants was necessary (or possible).
Blinding	There were no experiments for which blinding was necessary (or possible).

Did the study involve field work? ☐ Yes ☐ No

Field work, collection and transport

Field conditions	NA
Location	NA
Access & import/export	NA
Disturbance	NA

Reporting for specific materials, systems and methods

We require information from authors about some types of materials, experimental systems and methods used in many studies. Here, indicate whether each material, system or method listed is relevant to your study. If you are not sure if a list item applies to your research, read the appropriate section before selecting a response.

Materials & experimental systems

n/a	Involved in the study	
☒	☐	Antibodies
☒	☐	Eukaryotic cell lines
☒	☐	Palaeontology and archaeology
☒	☐	Animals and other organisms
☒	☐	Clinical data
☒	☐	Dual use research of concern
☒	☐	Plants

Methods

n/a	Involved in the study	
☒	☐	ChIP-seq
☒	☐	Flow cytometry
☒	☐	MRI-based neuroimaging

Plants

Seed stocks	NA
Novel plant genotypes	NA
Authentication	NA
